# Total Catch of a Red-Listed Marine Species Is an Order of Magnitude Higher than Official Data

**DOI:** 10.1371/journal.pone.0031216

**Published:** 2012-02-17

**Authors:** Alf Ring Kleiven, Esben Moland Olsen, Jon Helge Vølstad

**Affiliations:** 1 Institute of Marine Research (IMR), Arendal, Norway; 2 Faculty of Biosciences, Fisheries and Economics, University of Tromsø, Tromsø, Norway; 3 Centre for Ecological and Evolutionary Synthesis (CEES), Department of Biology, University of Oslo, Oslo, Norway; 4 Institute of Marine Research (IMR), Bergen, Norway; Swansea University, United Kingdom

## Abstract

Accurate information on total catch and effort is essential for successful fisheries management. Officially reported landings, however, may be underestimates of total catch in many fisheries. We investigated the fishery for the nationally red-listed European lobster (*Homarus gammarus*) in south-eastern Norway. Probability-based strip transect surveys were used to count buoys in the study area in combination with catch per unit effort data obtained independently from volunteer catch diaries, phone interviews, and questionnaires. We estimate that recreational catch accounts for 65% of total catch in the study area. Moreover, our results indicate that only a small proportion (24%) of lobsters landed commercially were sold through the legal market and documented. Total estimated lobster catch was nearly 14 times higher than reported officially. Our study highlights the need for adequate catch monitoring and data collection efforts in coastal areas, presents a clear warning to resource managers that illegal, unreported and unregulated (IUU) fisheries in coastal areas should not be ignored, and shows the potential impact of recreational fisheries.

## Introduction

Effective fisheries management depends on accurate estimates of total fishing effort and fishing mortality [1], [2], [3]. Pauly [4] argues, however, that official catch statistics generally are biased downward due to unreported commercial catch, and rarely accounting for small-scale and recreational fisheries. Indeed, when levels of unreported catch are evaluated, they tend to be significant [2], [5]–[8]. Assuming the level of unreported catch to be zero when in fact it is substantially higher, may jeopardize the sustainability of the resource concerned [7], [9].

Recently, the focus on illegal, unregulated and unreported (IUU) fishing [2], [7], [9]–[11] has increased. IUU fishing comprises a range of different legal and illegal activities, spanning from coastal areas to the high seas. Legal IUU activities can include recreational fisheries without catch statistics and commercial catches that agencies are not mandated to record or report. While, illegal activities might involve fishing in protected areas, non-compliance with regulations, and underreporting [2]. In general, IUU catches are difficult to estimate, and catch per unit effort (CPUE) from other fisheries, anecdotal information, and interviews are often used for the estimates [3].

Evidently, recreational fisheries can lead to declines in fish populations [12], and for certain populations may have higher catch levels than commercial fisheries [13]–[15]. In addition, catches taken by recreational fishers tend to include a large proportion of overfished species [14], [15]. Recreational fishers are a diverse group with different motivations for fishing [16], [17]; as a result it may be challenging to estimate their catch levels [18].

In this paper the fishery for European lobster (*Homarus gammarus*) serves as an example to investigate IUU fishing along the Norwegian coast. Lobster is a highly valued species targeted by both recreational and commercial fishers; who both have to follow the same regulations, except that the maximum number of traps allowed is higher for commercial fishers. Commercial fishers are registered in an official government fishery database, and are obliged to mark their lobster trap buoys with their registration number. Recreational fishers are not registered in any data base, but are obliged to write their name and address on their lobster trap buoys. Commercial CPUE has decreased in recent decades to historically low levels [19] and the lobster is now listed on Norway's red list as near threatened [20]. Catch statistics are available through mandatory official landings reports from the commercial fishing sector. In Norway, both commercial and recreational fisheries for lobster are open access and have no license requirements; neither quotas nor total effort regulations apply. Therefore, no information is available on the number of participants in the fishery from official records. Neither potential unreported commercial lobster catch nor recreational catch in the Norwegian lobster fisheries have been estimated before this study. We combine probability-based effort estimates [21] and CPUE data to estimate total catch in both commercial and recreational fisheries within season. We then compare these results with the official reported landings in the commercial lobster fishery.

## Materials and Methods

### Study Area

**Figure 1:**
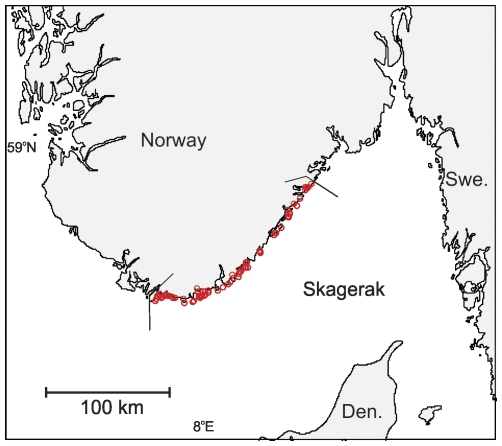
Study area. Distribution of fishing area for volunteer recreational fishers (red rings) that filled out catch diaries along Agder (Southern Skagerrak coast) in the 2008 fishing season. Angled lines indicate study area.

The study area was the Agder counties at the south-eastern Skagerrak coast of Norway, excluding areas west of the Lindesnes peninsula (Figure 1); the total coastal area between zero and 40 meters depth was 471.2 km^2^ [21]. In these counties, people live scattered along the coast and on islands, with boats docked on private properties and in small harbours. Coastal municipalities in the study area have a total of nearly 200,000 residents and also represent one of the most popular tourist destinations in the country.

### Estimates of Lobster Fishing Effort

Probability-based strip transect surveys were conducted to estimate lobster fishing effort (the average daily number of standing traps per week) within the study area during the 2008 lobster season (October–November). Surface buoys marking lobster traps were counted during week-long periods along strip transects placed randomly in the survey area. Transect width and detectability was calibrated through an experimental transect survey. The transect survey method is described in Kleiven et al. [21]. The transect surveys provided estimates of weekly effort for both recreational and commercial fishers throughout the season. Recreational fishers contributed to 66–70% of total effort during the first three weeks of the lobster season [21]. Later in the season recreational effort decreased relative to commercial effort (Table 1).

**Table 1:** Estimated number of total, recreational and commercial traps per day (1000) during the two-month lobster season (October–November) in 2008 for the study area in south Norway.

	Number of traps (1000)			
	*Total (1 SE)*	*Recreational*	*Commercial*	*Unknown*
Week 1	23.07 (1.47)	15.27	6.79	1.00
Week 2	22.06 (1.20)	15.51	5.97	0.58
Week 3	16.16 (1.42)	10.82	4.80	0.54
Week 4	11.69 (n/a)	7.33	3.91	0.46
Week 5	7.22 (0.63)	4.22	2.68	0.33
Week 6	5.75 (n/a)	3.17	2.37	0.22
Week 7	4.28 (n/a)	2.22	1.93	0.13
Week 8	2.81 (0.37)	1.36	1.38	0.06

Unknown is traps which could not be allocated to neither recreational nor commercial due to unclear registration on buoy. Three weeks (4, 6 and 7) were not surveyed and estimates are based on the surveys before and after and therefore lack variance estimate [21].

### Recreational Catch Diaries

Estimates of CPUE were obtained based on data provided by volunteer recreational fishers recruited by phone and at public meetings. Participants were asked to fill out a catch diary throughout the lobster season. A pilot survey with 15 recreational fishers filling out catch diaries was conducted in 2007. For every lobstering trip, the fishers were requested to provide information for each trap on the: soak time (the number of days gear was deployed); number of legal (≥25 cm total length) sized lobsters caught; number of sub-legal sized lobsters released; and number of egg-bearing females (which are protected) released. Fishers not returning the diary at the end of season were contacted by phone; they were asked if they had fished for lobster. If so, were encouraged to send in the diary. Fishers that had not completed the diary were asked whether or not they had fished during the season.

Recreational fishers that filled out diaries did not form a random sample. Hence, it was necessary to test if this group was representative of the total lobster fishing population in the study area. As part of the strip transects survey, Kleiven et al. [21] obtained fishers' contact information from a systematic random sample of buoys in the field; every fifth buoy observed within each transect, with a random start. Phone interviews of these fishers were conducted after the first week of lobster season to collect CPUE data for Aust-Agder (this eastern county representing approximately 50% of the total study area). At the end of the season, a mail-based questionnaire was sent to a randomly selected group of recreational fishers [21] to obtain information about : i) fishing periods; ii) numbers of traps deployed; iii) numbers of lobsters caught; iv) how often traps were hauled; and v) profile information, such as age and experience (years of lobster fishing). The same information was collected from fishers who filled out catch diaries.

We used a standard approach [22] to test if the estimated mean CPUE (legal sized lobsters per trap day^1^) based on data from the recruited diarists differed significantly from the estimated mean CPUE for the random selection of fishers:
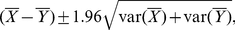
(i)where 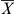 and 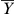 is the mean CPUE for the sample populations. CPUE for each respondent is calculated as a point estimate from which the mean and SE is calculated for the whole group; CPUE estimates are significantly different if the interval does not overlap zero.

### Commercial Fisheries

Selected commercial fishers have collaborated with the Institute of Marine Research (IMR) since 1928, providing CPUE data for the whole season [19]. These CPUE data are for scientific use only; reports provided by commercial fishers are treated confidentially and not shared with the management authorities. Note that these data provide only the mean CPUE (number of traps deployed, fishing time, and catch of legal sized lobsters) for the entire season. Therefore, data for the entire season was used to compare CPUE between recreational and commercial fishers. We estimated CPUE for each commercial and recreational fisher as
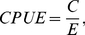
(ii)where C is total number of legal sized lobsters caught during the season, and E is total number of trap days for each fisher for the entire season. Due to the limited sample size of commercial fishers (n = 7 fishers) in the study area, we also did an extended comparison of CPUE for commercial and recreational fishers who kept catch diaries over a larger area (Norwegian part of Skagerrak). We used the standard method [22] to test for significant difference in CPUE between these groups (equation *i*).

Official landings data are collected and registered by the fishers own sales organization (Skagerakfisk), and registered as such. We acquired landings statistics (in kilos) from this organization: at each official landing facility in the study area. It was necessary to convert reported numbers of lobsters caught to weight, since official commercial landings are given in kilos. We used historical data (1963–1979) based on self-sampling by fishers in collaboration with IMR [19] in which each lobster in the sample (n = 5870) had weight and total length (measured from the tip of the rostrum to the posterior margin of the telson) estimated. In collaboration with a selection of commercial fishers, we also collected length data for lobsters landed during the 2008 season; every length measurement was also converted to weight based on historical length-weight data. We converted reported landings from kilos to number of lobsters to make the data comparable to recreational and commercial CPUE data collected as number of lobsters caught.

### Estimates of Lobster Total Catch

Total catch was estimated as the product of CPUE and E, which were both estimated from independent studies. Estimates of weekly catch are represented by the mean CPUE as lobsters per trap week^−1^. The first week was defined as the seven days following the opening of lobster season. Let 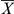 and 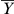 denote mean CPUE and E, respectively. An estimator of total catch is then the product
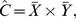
(ii)Since CPUE and E were estimated in separate studies, X and Y can be considered as independent variables. Hence, an unbiased estimator for the variance of 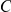 is given as [23]:
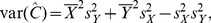
(iii)where 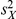 and 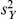 are the variances of mean CPUE and E, respectively.

## Results

### Catch Rate Data Collection

A total of 106 catch diaries were sent out to individual recruited fishers, of which 77 diaries were returned. Follow-up phone interviews revealed that nine persons had not participated in the lobster fishery; while 20 persons had participated in the lobster fishery, but had not reported catch in the diary. Therefore, the diary response rate for fishers that took part in lobster fishery was estimated to be 79%. Sixty two of those returning catch diaries also filled out an additional questionnaire designed to determine their age and experience.

### Test of Representativity

We tested the assumption that recreational fishers who participated in the diary survey formed a representative sample of all lobster fishers in the area. The group of fishers providing diaries and those included in the random sample were comparable (i.e. no significant difference) with regard to age composition, experience (years of lobster fishing), number of active fishing days and time between trap hauls (Table 2). Moreover, we did not find a significant difference in CPUE between the random selection of fishers registered in the field (n = 24, CPUE = 0.118, SE = 0.017) and those who filled out catch diaries (n = 35, CPUE = 0.112, SE = 0.015) during the first week of the season in the eastern half of the study area. We therefore conclude that fishers recruited to fill out diaries can be considered a representative sample of the recreational lobster fishing population, and that the diaries provide representative estimates of CPUE that can be used in conjunction with effort estimates to estimate total recreational catch.

**Table 2:** Test of the representativity of volunteer recreational catch diaries as i) between recreational diarists and a random sample of recreational fishers and ii) between recreational diarists and commercial fishers.

		Diary		Test pop			
		Mean	SE	Mean	SE	Interval	Sig
i) Whole season Diary and random questionnaire within study area	CPUE	0.064	0.006	0.060	0.005	0.02, −0.01	no
	YB	1953.8	1.7	1952.2	2.5	6.7, −5.1	no
	FY	24.5	2.0	23.9	2.9	8.4, −5.4	no
	FD	34.1	2.1	30.9	2.8	10.1, −3.7	no
	TH	2.17	0.09	2.21	0.19	0.4, −0.5	no
i) First week, diary and random phone interview Aust-Agder		0.112	0.007	0.118	0.017	0.03, −0.04	no
*ii)* Whole season, Diary and commercial fishers within study area		0.064	0.006	0.069	0.020	0.04, −0.05	no
*ii*) Whole season. Dairy and commercial fishers in Skagerrak		0.066	0.005	0.070	0.009	0.02, −0.02	no

CPUE is lobsters trap^−1^ day^−1^.

Mean year born (YB), years of lobster fishing experience (FY), fishing days (FD) and time between each trap haul (TH), incl. mean S.E. for the test sample and recruited diary reporters. The first week of the season (Aust-Agder, eastern part of the survey area) and for mean of season (whole study area) for diary and a random selection of fishers registered in field. If the interval for the difference contains zero, it is no significant difference [32].

We tested if weekly mean CPUE from catch diaries provided by recreational fishers could be considered representative of the mean CPUE in the commercial fishery (Table 2). CPUE for the whole season was therefore tested between the recreational lobster diarists and the reported commercial CPUE for the study area. CPUE for the small sample of commercial fishers (n = 7) in the study area was 0.069 (SE = 0.015). The mean number of active fishing days for the study area during the two month open season for commercial fishers was 33.6 days, while recreational fishers had a mean of 34.1 fishing days. To test the general assumption of no difference in CPUE between commercial and recreational fishers, we compared all recreational lobster diarists from the Norwegian part of Skagerrak (n = 95, CPUE = 0.066, SE = 0.005) with the commercial catch rate for the same region (n = 21, CPUE = 0.070, SE = 0.008). Even though commercial catch rates tend to be slightly higher than recreational fishers, the difference between the two groups was not significant. We conclude that mean CPUEs from recreational catch diaries could be combined with independent effort estimates for commercial fishers to estimate total commercial catch. Resulting catch estimates may be slightly biased downwards if commercial fishers have higher catch rates than recreational fishers.

### Weight-length Relationships and Reported Landings

Mean weight of legal lobsters was estimated to be 653 grams (n = 837, SE = 7). Total official commercial landings for the study area were 1,813 kilos (Skagerakfisk landing statistics 2009).

### Total Catch in the Fishery

**Figure 2:**
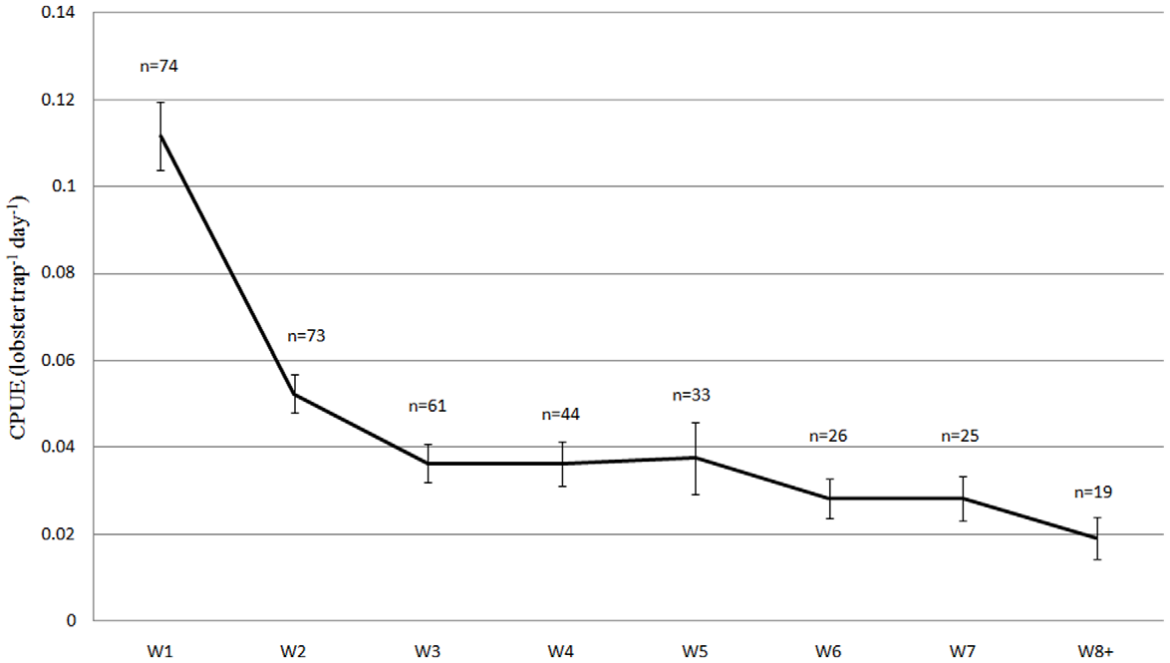
Weekly catch rate. Mean CPUE (lobster trap^−1^ day^−1^) for each week through the lobster season from catch diaries. Error bars indicate SE of mean. n = number of catch diaries for the given week.

**Figure 3:**
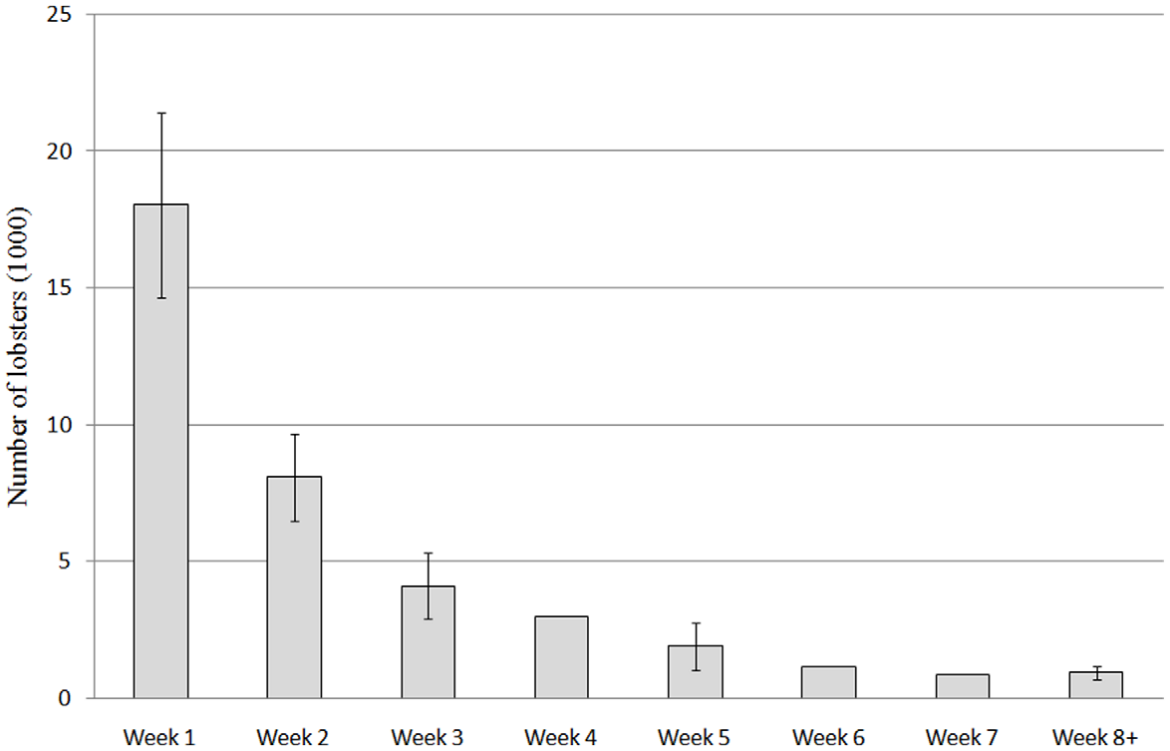
Total catch. Total weekly catch (number of lobsters) in the lobster fishery 2008 in the study area. Error bars indicate 95% CI. The catch is highest in the first week of the season.

CPUE was highest during the first week of the season, and then decreased by nearly 50% during the second week (Figure 2). By end of the fourth week, CPUE had decreased further to 17% of what was observed during the first week. Catches were highest during the first week of the season, accounting for 47% of estimated total landings. Seventy-seven percent of the lobsters were landed during the three first weeks (Figure 3). Recreational catch accounted for 65% of total catch, while commercial catch accounted for only 31% of the total (Table 3). Four percent of the catch could not be allocated to either recreational or commercial sector, since the traps were either unmarked or unreadable [21].

**Table 3:** Total estimated landings of lobster in numbers and kilos for recreational, commercial and official data.

	# of lobsters	Kilo
Total recreational catch	24900	16200
Total commercial catch	11700	7700
Total unknown catch	1400	900
Total catch	38000	24800
Official landing data	2800	1813

Unknown catch is lobster caught by fishing gear that could not be allocated to recreational nor commercial sector, since the buoys were unmarked or unreadable.

## Discussion

This study demonstrates that official landings data reported in Norway for European lobster (a red-listed marine species) dramatically underestimate the total catch due to significant levels of unreported catch from both commercial and recreational fisheries. We found that official landings represent just 24% of the total estimated commercial catch. For combined commercial and recreational fisheries, our estimates of catch are nearly 14 times higher than the official landings data. Commercial fishers are legally obliged to report their lobster catch; not doing so qualifies as IUU fishing due to underreporting or not reporting catch. The amount of unreported catch exceeds official landings to such an extent that official landings statistics appear unreliable. This implies that official landings statistics for lobsters should not be used in stock assessment or to set quotas. Norway has a good reputation for implementing ecosystem- based management and adherence to the FAO code-of-conduct for responsible fisheries [24], [25]. Substantial Norwegian resources have been used to combat IUU fishing on shared international fish stocks [26]. The Norwegian Ministry of Fisheries and Coastal Affairs [27] states that: “Illegal, unreported and unregulated fishing (IUU fishing) is one of the most serious problems currently facing the management of the world's fisheries. Both legal harvesting and marine ecosystems are threatened. Fighting this crime is the highest priority of Norwegian fisheries management”. It can be argued that Norwegian coastal fisheries, such as the fishery for European lobster, have not been a major focus in developing the strategy to combat IUU fishing. Furthermore, IUU fishing could also affect total catch for other coastal species under the same management regime. Our results show that even advanced fishing nations may have serious challenges to control and manage use of coastal resources. When total catch of a red listed species appears to be an order of magnitude higher than officially reported, clearly there is a need to re-evaluate the system for collecting catch data, and practices for fisheries management and surveillance in coastal Norway.

We believe that our findings represent the highest proportion of removals for any recreational lobster fishery described to date, with a catch level that is twice that of the commercial catch. Significant effort and catch by recreational lobster fishers is also observed in Australia [28], the US [29] and South Africa [30].

Catch rates of commercial and recreational fishers were comparable. CPUE of commercial fishers estimated from the standardised long-term time series of data collected by IMR is not necessarily representative, since the group of fishers was not randomly selected. This could cause bias since fishers reporting their catch rates to IMR may not be representative for the entire commercial lobster fishing sector in the area. However, differences in catch rates between groups studied (both recreational and commercial fishers) are insignificant, and the low variation between fishers within these groups suggest that any expected bias would be low.

Information from questionnaires sent to all commercial fishers observed in the study area was treated anonymously. This was a necessary precaution since many of the potential respondents may have underreported their catch to the management authorities. As a consequence, a follow-up survey of non-respondents could not be conducted. However, the information gathered from the questionnaire was related to additional information about the number of traps per buoy and other gear types used in the lobster fishing season, and was not used to estimate CPUE from this sector. We recognize that the sample size of commercial fishers in the study area was low (n = 7). However, a test of CPUE between commercial and recreational fishers for a larger area (Skagerrak) supports the assumption that the catch rate is not significantly different between these two groups.

Our estimate of total catch is likely to be conservative. Kleiven et al. [21] calculated that the proportion of traps deeper than 40 meters represented 2.8% of the total effort. Fishing effort from these traps was not included in the estimate of total effort; subsequently it was not included in the estimate of total catch. Further, Kleiven et al. [21] argues that effort estimates from the strip transect surveys are likely to be underestimates at the beginning of the season and overestimates at the end. The reason being that a standardised transect width calculated by calibration studies was used throughout the season, even though mean transect width appeared to increase slightly through the survey period. This could lead to underestimates of total catch since the highest effort and catch rates were observed at the beginning of the season. In addition, a small proportion (4%) of the traps were attached to buoys that were either unmarked or had markings that were illegible. This effort was included in the total catch estimate, but not assigned to neither commercial nor recreational fishers. Including these observations as either recreational or commercial could lead to a bias if one of the groups has a higher frequency of wrongly marked buoys. Our estimates relate to the lobsters caught within the legal fishing season. It is suspected that lobsters are caught outside the legal season. Lobster fishing mortality outside the legal fishing season would further increase the total IUU catch.

Through the use of probability-based strip transects to estimate effort and using catch diaries to estimate CPUE, we achieved a high level of precision in the total catch estimate. However, the method used is time consuming, costly, and weather dependent. A system for licensing recreational fishers, as used in Australia [26], the US [27], and South-Africa [28] would reduce costs and be more effective to estimate recreational landings.

The lack of total effort and/or catch regulations may preclude a recovery of the lobster population, even though other regulations have been implemented. A small increase in CPUE may lead to increased participation in the fishery that has the potential to counteract efforts to rebuild the lobster population. Our results indicate that there is a ‘race to fish’ where both CPUE and effort are highest during the first days of the season. Further research is needed to investigate the relationship between the population size and the rapid decrease in CPUE.

While offshore fisheries typically are dominated by larger commercial vessels, the coastal fishery consists of recreational and small-scale commercial fishers. Due to this complexity, total catch in coastal fisheries is a challenge to estimate, and often ignored by management authorities [31]. Collecting reliable catch data is therefore essential for effective resource management, and should include ensure that commercial landings are reported accurately. Doing so would require increased effort to guarantee compliance, and better surveillance of lobsters as they are marketed and sold. Further, a data collection or catch estimation framework for all recreational fisheries should be established nationally. Furthermore, regulation of total effort and total catch in the fishery is needed to rebuild the red-listed European lobster in Norway. Also, developing a network of lobster reserves would have the potential to protect components of the lobster population from the intense fishing pressure [32].

The lobster fishery in Norway is small relative to other coastal fisheries, which speaks to an urgent need to evaluate IUU removals and recreational fisheries for a number of other valuable coastal species such as cod (*Gadus morhua*), halibut (*Hippoglossus hippoglossus*), ling (*Molva molva*) and norway lobster (*Nephros norvegicus*), which are also popular recreational target species. Our results highlight the need for appropriate data collection systems for catch in Norwegian coastal areas. It also serves to warn management authorities of the consequences of ignoring coastal IUU fisheries, and the potential impact of recreational fisheries.
